# Vital Pulp Therapy of a Mature Molar with Concurrent Hyperplastic Pulpitis, Internal Root Resorption and Periradicular Periodontitis: A Case Report

**DOI:** 10.7508/iej.2015.03.015

**Published:** 2015

**Authors:** Saeed Asgary, Mehmet Kemal Çalışkan

**Affiliations:** a * Iranian Center for Endodontic Research, Research Institute of Dental Sciences, Dental School, Shahid Beheshti University of Medical Sciences, Tehran, Iran;*; b *Department of Endodontics, Dental School, Ege University, İzmir, Turkey*

**Keywords:** Apical Periodontitis, Calcium-Enriched Mixture, Endodontic, Hyperplastic Pulpitis, Irreversible Pulpitis, Permanent Teeth, Pulp Polyp, Pulpotomy, Root Resorption, Vital Pulp Therapy

## Abstract

Vital pulp therapy (VPT) of permanent mature teeth is continuously ascertaining to be a more reliable endodontic treatment. The purpose of this case report was to describe successful VPT of a mature mandibular left first molar with concurrent hyperplastic pulpitis, internal root resorption and periradicular periodontitis in a 35-year-old male patient. After complete caries removal and access cavity preparation, the dental pulp was removed from the coronal third of the roots. To protect the remaining pulp, calcium-enriched mixture (CEM) cement was placed and adapted into the cavities; the tooth was then restored with amalgam. Six months after VPT, radiographic examination showed evidence of periradicular healing. Clinically, the tooth was functional without signs and symptoms of infection/inflammation. The successful outcome of this case suggests that diseased dental pulp (*i.e*. irreversible pulpitis) has the potential to heal after pulp protection with CEM biocement.

## Introduction

*Hyperplastic pulpitis*, *aka* pulp polyp is the chronic inflammation of carious/traumatic exposed pulp in deciduous or newly erupted permanent teeth. This condition is classified as asymptomatic irreversible pulpitis and treatment of the involved tooth includes removal of the polyp, pulpectomy and root canal therapy (RCT) [1]. However, since 1951 several successful vital pulp therapies (VPTs) for various teeth with hyperplastic pulpitis have been reported [2-4].

Internal root resorption (IRR) which is usually diagnosed upon routine intraoral radiographic examination is the progressive destruction of root canal walls as a result of increased odontoclastic activity. The etiology of IRR is not clear, however, chronic pulpitis and dental trauma are considered to be the main risk factors; this condition is also classified as asymptomatic irreversible pulpitis. RCT is the treatment of choice as it removes the blood supply of the odontoclasts [5]. Recently, a novel treatment option for teeth with IRR has been reported *via* application of calcium hydroxide in the coronal side of the lesion [6, 7].

Apical periodontitis (AP) is the inflammation of the periodontium around the communication portals of an infected root canal system in necrotic teeth, however, it also may exist in vital teeth with severely inflamed pulps (*i.e.* irreversible pulpitis) [8]. Epidemiological studies have publicized that healing of AP in vital/non-vital teeth depends on the quality of RCT as well as the coronal seal [9]. However, recent randomized clinical trials have revealed that in molar teeth with irreversible pulpitis associated with apical periodontitis, VPT produced more success at 6- and 12-month recalls compared to RCT [10].

Calcium-enriched mixture (CEM) cement is a bioceramic used in a variety of clinical applications; one-step apexification or revascularization treatment for necrotic immature permanent teeth, management of internal/external root resorption, repair of furcal/root perforations, root-end fillings and pulp protection in various VPTs for primary/permanent teeth [11, 12]. 

**Figure 1:**
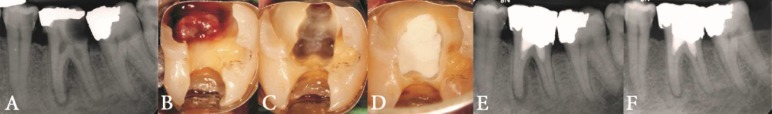
*A)* Preoperative radiophotograph showing extensive caries and internal root resorption in coronal part of mesial/distal canals in a mandibular left first molar; *B) *Clinical photograph after removing the restoration, *C)* Clinical photograph after pulp tissue removal and hemostasis, *D)* Clinical photograph after placement of CEM cement, *E)* Postoperative radiophotography showing placement of CEM in the prepared cavities and amalgam restoration, *F)* Six-month follow-up showing establishment of periodontal ligament space and lamina-dura

This case presented successful outcomes after VPT of a concurrent hyperplastic pulpitis, internal root resorption and periradicular periodontitis in a mature mandibular molar using CEM cement after a six-month recall.

## Case Report

A 35 year-old male with a symptomatic mandibular first left molar was assessed at a private endodontic clinic. He complained of mild pain during chewing. Clinical examination showed an old amalgam restoration with extensive recurrent caries in the first molar. The tooth was slightly sensitive to percussion but not to palpation. Probing elicited moderate pain and consequent bleeding that indicated a pulp polyp. Radiographic examination demonstrated an amalgam-restored tooth with an extensive cavity; two large radiolucent lesions located at the inner coronal part of both distal and mesial root canals and noticeable periradicular radiolucencies surrounding the mesial and distal root apices (Figure 1A). Patient’s medical history revealed no significant findings. Considering the clinical and radiographic findings, our concluding diagnosis was internal root resorption and hyperplastic pulpitis (asymptomatic irreversible pulpitis) associated with periradicular periodontitis. Vital pulp therapy (VPT) was decided to be performed. After explanation of possible risks of VPT, written informed consent was obtained from the patient.

After mouth rinse with 0.2% chlorhexidine, the tooth was anesthetized with 2% lidocaine with 1:100000 epinephrine (Dentsply Pharmaceutical, PA, USA) and then isolated. Subsequently, the amalgam restoration was removed, tooth decay was excavated (pulp polyp was completely visible; Figure 1B), and canal orifices were then accessed using a diamond-coated fissure bur (Diatech, Heerbrugg, Switzerland). The coronal part of the root canals was prepared using #2 to 6 Gates-Glidden burs (Dentsply Maillefer, Ballaigues, Switzerland) and copious irrigation. After 3 min, hemorrhage was controlled with sterile cotton pellets soaked in chlorhexidine and placed in the chamber. Once the pulpal tissues were clot free with no bleeding (Figure 1C), CEM cement powder and liquid (BioniqueDent, Tehran, Iran) were mixed according to the manufacturer’s instructions and delivered to the pulp chamber using a sterile plastic instrument. The biocement was gently adapted to the coronal portion of the canals using sterile dry cotton pellet and pre-fitted endodontic pluggers (M-series, Dentsply Maillefer, Tulsa, USA) (Figure 1D). Finally, the tooth was restored permanently with amalgam (SDI gs80, SDI limited, Australia) (Figure 1E). 

The patient was recalled one week after treatment. Clinical examinations at this time showed that the tooth was not sensitive to percussion/palpation, and the patient did not have any complaint about chewing with the tooth. At six-month recall, the patient reported no signs or symptoms. Clinical examination showed a functional tooth, which was symptom-free, with normal physiologic mobility, normal probing depths and a satisfactory coronal restoration. Intraoral radiographic examinations revealed normal periodontium and evidence of periradicular healing (Figure 1F).

## Discussion

This report represented a successful VPT procedure in a vital molar tooth with IRR and hyperplastic pulpitis associated with periradicular lesions. After 6 months, the preoperative periradicular lesions had resolved with re-establishment of a normal PDL space and lamina-dura. 

Internal and external root resorptions are often confused/ misdiagnosed. In this case, based on pretreatment radiographic findings such as clearly defined margins, uniform density and ballooning out of root canal walls, radiolucencies in the coronal aspect of both mesial and distal roots were diagnosed as IRR. Universally, to arrest the progression of IRR, the treatment options include pulpectomy and root filling with traditional or new endodontic bio/materials. As radiographic and histologic studies have indicated that lesions of internal resorption may cease and dentin formation may recur, researchers have suggested pulpotomy with CEM/calcium hydroxide as a successful treatment for IRR [6, 13]. However, in the present case, dental pulp was removed from the pulp chamber as well as resorptive defects and the remaining radicular pulp was protected with CEM biomaterial. 

Clinicians have a preference to extirpate an entire diseased pulp; however, a growing body of evidence from recent clinical trials as the current best evidence has publicized that mature teeth with irreversible pulpitis can be treated effectively by VPT. Therefore, justification for the novel VPT technique is based on the healing potential of pulpal tissue (which encompass stem cells) and also the biocompatibility of pulp covering agents [10, 14]. 

There is a direct cause-and-effect relationship between microorganisms/their by-products and inflammation of the pulp/periradicular tissues. Therefore, an ideal endodontic treatment should focus on elimination of etiologic factors as well as protecting pulpal tissue with a biocompatible, antibacterial and sealable material to provide a suitable condition for pulp healing. One-year results of a recent randomized clinical trial have revealed that in mature vital molars with irreversible pulpitis associated with apical involvement, VPT using CEM cement created favorable conditions for periapical healing in ~91% of cases [10]; the findings of the present case were also consistent with the trial and previous reports [15-17].

In the present case, protrusion of granulation tissue from the pulp chamber that bled readily on probing enabled the diagnosis of an ulcerated hyperplastic pulpitis. In hyperplastic pulpitis, the tissue in the pulp chamber is frequently transformed into granulation tissue; however, radicular pulp tissue may remain normal/vital with vasodilatation [18]; undoubtedly, virtuous blood supply facilitates pulpal defense and healing. Teeth with hyperplastic pulpitis are generally treated by RCT; however, VPT procedures have been recommended to treat such teeth [4, 19].

In this case CEM cement was selected because it has many favorable properties, which include biocompatibility, antibacterial/fungal effects, good sealing capacity, ability to set in the presence of moisture and to induce hard tissue formation *i.e.* osteogenesis, dentinogenesis and cementogenesis [11, 14].

## Conclusion

In the present case, clinical and radiographic evidence showed successful use of CEM cement for VPT of a mature permanent molar with IRR and hyperplastic pulpitis associated with periradicular lesion. Further large-scale clinical trials with long-term follow-up periods are necessary to confirm this protocol.
